# Supplementary material for: Detailed molecular and epigenetic characterization of the pig IPEC-J2 and chicken SL-29 cell lines
Source: iScience. 2023 Feb 20;26(3):106252. doi: 10.1016/j.isci.2023.106252 (PMC10018572; doi:10.1016/j.isci.2023.106252)
Supplement: Data S2. Complete homer output for identified motifs in Chicken SL-29, related to Tables 5 and 6 — Homer motif analysis results for histone modifications H3K4me1, H3K4me3, H3K27ac, enhancers, and ATAC-seq of chicken SL-29 cell line. Parameters for possible false positives is as mentioned earlier for S5. [file mmc3.zip › Data_S2/S6/Chicken_SL_29/peak_files_CTCF/homerResults/motif12.similar.html]

motif12

## Information for motif12

A
G
T
C
T
C
A
G
T
C
A
G
A
T
G
C
A
G
T
C
T
G
C
A
C
T
G
A
A
C
G
T
T
G
A
C
T
C
A
G
  
Reverse Opposite:  

A
G
T
C
A
C
T
G
T
G
C
A
A
G
C
T
A
C
G
T
A
C
T
G
A
T
C
G
A
G
T
C
A
G
T
C
T
C
A
G
  

|  |  |
| --- | --- |
| p-value: | 1e-63 |
| log p-value: | -1.464e+02 |
| Information Content per bp: | 1.631 |
| Number of Target Sequences with motif | 1363.0 |
| Percentage of Target Sequences with motif | 4.62% |
| Number of Background Sequences with motif | 827.2 |
| Percentage of Background Sequences with motif | 2.84% |
| Average Position of motif in Targets | 148.5 +/- 81.9bp |
| Average Position of motif in Background | 156.0 +/- 92.2bp |
| Strand Bias (log2 ratio + to - strand density) | -0.1 |
| Multiplicity (# of sites on avg that occur together) | 1.34 |
| Motif File: | file (matrix) reverse opposite |

### Similar de novo motifs found

|  |  |  |  |  |  |  |  |
| --- | --- | --- | --- | --- | --- | --- | --- |
| Rank | Match Score | Redundant Motif | P-value | log P-value | % of Targets | % of Background | Motif file |
| 1 | 0.729 | A T C G G T A C A T C G T A C G G T A C A G T C G C T A C G T A | 1e-58 | -135.193556 | 15.82% | 12.58% | motif file (matrix) |
| 2 | 0.922 | T C A G A T G C A G T C C G T A C T G A C G A T T G A C T C A G | 1e-57 | -132.936429 | 2.81% | 1.53% | motif file (matrix) |
| 3 | 0.848 | G A T C A G T C C G T A G T C A C G A T T A G C T C A G T A C G T C G A C T A G T A C G A T C G T A G C | 1e-51 | -118.215516 | 2.96% | 1.70% | motif file (matrix) |
